# Liraglutide Improves the Angiogenic Capability of EPC and Promotes Ischemic Angiogenesis in Mice under Diabetic Conditions through an Nrf2-Dependent Mechanism

**DOI:** 10.3390/cells11233821

**Published:** 2022-11-29

**Authors:** Xiaoqing Yan, Yue Su, Xia Fan, Hui Chen, Zixian Lu, Zijuan Liu, Yingjian Li, Mei Yi, Guigui Zhang, Chunjie Gu, Kai Wang, Jiamin Wu, Da Sun, Yikai Zhang, Chi Zhang, Xiaozhen Dai, Chao Zheng

**Affiliations:** 1 Chinese-American Research Institute for Diabetic Complications, School of Pharmaceutical Sciences, Wenzhou Medical University, Wenzhou 325035, China; 2 The Second School of Medicine, Wenzhou Medical University, Wenzhou 325027, China; 3 The Third Affiliated Hospital of Wenzhou Medical University, Wenzhou 325200, China; 4 Department of Pediatrics, Endocrinology and Metabolism, The First Affiliated Hospital of Wenzhou Medical University, Wenzhou 325200, China; 5 Institute of Life Sciences, Wenzhou University, Wenzhou 325200, China; 6 Department of Endocrinology, The Second Affiliated Hospital, School of Medicine, Zhejiang University, Hangzhou 310058, China; 7 School of Biosciences and Technology, Chengdu Medical College, Chengdu 610500, China

**Keywords:** liraglutide, diabetes, endothelial progenitor cells, angiogenesis, Nrf2

## Abstract

The impairment in endothelial progenitor cell (EPC) functions results in dysregulation of vascular homeostasis and dysfunction of the endothelium under diabetic conditions. Improving EPC function has been considered as a promising strategy for ameliorating diabetic vascular complications. Liraglutide has been widely used as a therapeutic agent for diabetes. However, the effects and mechanisms of liraglutide on EPC dysfunction remain unclear. The capability of liraglutide in promoting blood perfusion and angiogenesis under diabetic conditions was evaluated in the hind limb ischemia model of diabetic mice. The effect of liraglutide on the angiogenic function of EPC was evaluated by cell scratch recovery assay, tube formation assay, and nitric oxide production. RNA sequencing was performed to assess the underlying mechanisms. Liraglutide enhanced blood perfusion and angiogenesis in the ischemic hindlimb of db/db mice and streptozotocin-induced type 1 diabetic mice. Additionally, liraglutide improved tube formation, cell migration, and nitric oxide production of high glucose (HG)-treated EPC. Assessment of liraglutide target pathways revealed a network of genes involved in antioxidant activity. Further mechanism study showed that liraglutide decreased the production of reactive oxygen species and increased the activity of nuclear factor erythroid 2-related factor 2 (Nrf2). Nrf2 deficiency attenuated the beneficial effects of liraglutide on improving EPC function and promoting ischemic angiogenesis under diabetic conditions. Moreover, liraglutide activates Nrf2 through an AKT/GSK3β/Fyn pathway, and inhibiting this pathway abolished liraglutide-induced Nrf2 activation and EPC function improvement. Overall, these results suggest that Liraglutide represents therapeutic potential in promoting EPC function and ameliorating ischemic angiogenesis under diabetic conditions, and these beneficial effects relied on Nrf2 activation.

## 1. Introduction

Microvascular and macrovascular complications of diabetes, including diabetic cardiovascular diseases, diabetic retinopathy, and diabetic foot, are the leading causes of morbidity and mortality in patients with diabetes. These vascular complications are associated with dysregulation of vascular homeostasis and dysfunction of the endothelium under diabetic conditions, which implies that approaches promoting vascular function and ameliorating angiogenesis are promising therapeutic strategies for diabetic vascular complications [1]. Endothelial progenitor cells (EPC) are a class of precursors of endothelial cells that play pivotal roles in maintaining endothelial homeostasis [2] and triggering angiogenesis [3]. EPC promote angiogenesis through directly incorporating into ischemic tissues to form neo-vessels [4,5] and/or by secreting proangiogenic factors [6]. The number and function of EPC are inversely correlated with coronary artery disease [7] and the cumulative cardiovascular risk [8]. Transplanting with EPC from cord blood [9], peripheral blood [10], or bone marrow [11] can promote ischemic angiogenesis and improves the function of ischemic tissue in animals with myocardial or hindlimb ischemia. However, the number of circulating EPC is decreased, and their function is impaired in subjects with type 1 diabetes (T1DM) [12] or type 2 diabetes (T2DM) [13], which impairs endothelial homeostasis and attenuates angiogenesis. Thus, improving EPC function and/or increasing EPC number has been considered as a promising strategy for the amelioration of diabetic vascular complications.

Glucagon-like peptide-1 (GLP-1) is a brain-gut peptide produced predominantly in enteroendocrine cells [14]. GLP-1 controls glucose metabolism and energy homeostasis by regulating food intake, islet hormone secretion and gastrointestinal motility via GLP-1 receptor (GLP-1R), enabling the development of GLP-1R agonists for the treatment of diabetes and obesity [15]. Liraglutide, a GLP-1 analogue, is a first-line medication forT2DM. Liraglutide aids in glycemic control by repressing appetite, reducing glucagon secretion, and stimulating postprandial release of insulin [16]. In addition to its antidiabetic activity, recent studies have revealed the cardioprotective and vascular protective effects of liraglutide [17]. Clinical trial showed that liraglutide reduces the risk of major cardiovascular events and all-cause mortality in T2DM patients [18,19,20,21]. However, the effect and mechanism of liraglutide on diabetic angiogenesis is largely unknown.

In the current study, we aimed to investigate whether liraglutide ameliorates ischemic angiogenesis under diabetic conditions, rescues EPC from diabetes-induced dysfunction, and further reveal the underlying mechanisms.

## 2. Materials and Methods

### 2.1. Animals

Twelve-week-old male db/db mice (BKS-Leprem2Cd479/Gpt, GemPharmatech, Nanjing, China) were used as a T2DM mouse model. Male C57BL/6JGpt mice (GemPharmatech) and nuclear factor erythroid 2-related factor 2 knockout (Nrf2-KO) mice (kindly provided by Prof. Xuebo Pan at Wenzhou Medical University) aged 8–12 weeks were used to establish T1DM model by streptozotocin (STZ, Sigma, St. Louis, MO, USA) injection (50 mg/kg body weight, one dose a day for 5 days). Those mice with a fasting blood glucose higher than 13.8 mM at the 7th day after the last STZ injection were considered as T1DM mice. Mice were maintained at 22 ± 2 °C with a 12 h light/dark cycle. All animal experimental procedures were carried out in accordance with the policies of Institutional Animal Care and the Use Committee of Wenzhou Medical University.

### 2.2. Hindlimb Ischemia Model Construction and Liraglutide Administration

Hindlimb ischemia (HLI) model was constructed in both T2DM mice and T1DM mice as previously described [22]. Briefly, after sufficient anesthesia with isoflurane (RWD, Shenzhen, China. 1–3% isoflurane in 100% oxygen, 1 L/min), the hindlimbs of the mice were shaved and dissection was performed on the right hindlimb. Then, the superficial femoral artery was double-ligated and subsequently cut off. Finally, the incision was stitched up layer by layer. Diabetic HLI mice were randomly divided into two groups, the liraglutide group and vehicle group. The liraglutide group was pretreated with liraglutide (200 µg/kg body weight, daily) via subcutaneous injection for 2 days before HLI surgery, and continually treated for an additional 28 days until the mice were terminated. The vehicle group was treated with an equivalent volume of normal saline. During this period, blood perfusion of ischemic hindlimbs was monitored. Mice were terminated on the 28th day post-surgery, and plasma samples and gastrocnemius muscle tissues were collected for further assays.

### 2.3. Blood Perfusion Scanning

To monitor the blood perfusion recovery of ischemic hindlimbs, blood perfusion scanning was performed before surgery and on day 0, 3, 7, 14, 21 and 28 after surgery using a PeriCam Perfusion Speckle Imager (PSI, Perimed, Kings Park, NY, USA) as previously described [23]. Briefly, after sufficient anesthesia with isoflurane, mice were placed in a prone position and the anal temperature of mice was maintained around 37 °C, and blood perfusion of both hindlimbs was detected according to the manufacturer’s instructions. 

The blood perfusion in the nonischemic hindlimb of each mouse was used as a self-control, and the blood perfusion of the ischemic hindlimb was presented as the ratio of blood flow in the ischemic limb (right) to that in the non-ischemic limb (left).

### 2.4. Immunofluorescence Staining

The extent of angiogenesis in ischemic hindlimbs was evaluated by the capillary density through CD31 and dystrophin staining (to indicate myofibers). Briefly, frozen sections (6 μm) of gastrocnemius muscles in the ischemic hindlimbs were fixed with cold methanol for 15 min. After three washes with PBS, sections were blocked for 1 h using a blocking buffer (PBS containing 5% goat serum). Thereafter, sections were incubated with a primary antibody against dystrophin (Proteintech, Wuhan, China) at 4 °C overnight. After being washed three times with PBS, sections were incubated with the corresponding PE-conjugated secondary antibody (Cell Signaling Technology, Boston, MA, USA) and the FITC-conjugated anti-CD31 primary antibody (BD Biosciences, Franklin, MA, USA) at room temperature in the dark for 1 h, and then washed. The sections were sealed, and images were captured using a fluorescence microscope (Olympus IX71, Olympus, Tokyo, Japan). The number of capillaries per myofibers was calculated in randomly selected fields for a total of 10 different fields per section and three sections per mouse. The capillary density was reported as the number of CD31-positive capillaries per myofiber.

### 2.5. EPC Isolation and Identification

EPC were isolated from human umbilical cord blood by density gradient centrifugation as previously described [4,5]. The Institutional Review Board of The First Affiliated Hospital of Wenzhou Medical University approved all protocols, and informed consent was obtained. Briefly, anticoagulant cord blood (50–100 mL) was diluted 1:1 with Hanks balanced salt solution (HBSS; Invitrogen, Grand Island, NY, USA), carefully overlaid onto an equivalent volume of Histopaque 1077 (Sigma) and centrifuged at 400 g for 30 min at room temperature. Thereafter, the buffy coat was collected and washed twice with HBSS. Cells were resuspended in EGM-2 supplemented with 2% FBS (Lonza, Basel, Switzerland) and cultured in one well of a six-well plate that was precoated with fibronectin (2 μg/cm^2^, BD Biosciences). The cells were cultured at 37 °C in a humidified environment with 5% CO_2_. After 24 h, unattached cells and debris were removed by gentle washing. The medium was changed daily for 7 days and thereafter on alternate days. Clones appeared between 14–21 days and reached 80% confluence at approximately 28 days. After subculture, cell surface markers CD34, VE-cadherin (CD144), VEGFR2, CD14, and CD45 were detected by flow cytometry using corresponding antibodies (BD Biosciences) to characterize EPC. EPC were expanded to the fourth or fifth passage for further analysis.

### 2.6. Cell Scratch Recovery Assay

The migration capability of EPC was investigated using cell scratch recovery assay. EPC (1 × 10^5^ cells per well) were seeded onto 24-well plates and grown to confluence. Thereafter, a scratch was made in each well using a T1000 tip. Remaining EPC were cultured in MCDB131 medium containing an additional 19.5 mM of glucose (final concentration of glucose is 25 mM, high glucose, HG) or 19.5 mM of mannitol (Man), along with liraglutide (3, 6 or 12 μg/mL) or an equivalent volume of PBS. The cells were cultured at 37 °C in a humidified environment with 5% CO_2_ for 24 h. Mitomycin (1 μg/mL, Sigma) was added to each well to exclude the effect of cell proliferation. Images of scratches were captured in randomly selected high magnification fields (10×) in each well under a light microscope (DMI3000B; Leica, Wetzlar, Germany) equipped with a digital camera (Olympus DP25). The area of scratch wound was measured using ImageJ (“http://rsbweb.nih.gov/ij/”, accessed on 23 February 2020), and scratch wound recovery percentage equals ([Scratch area at 0 h]–[Scratch area at 24 h])/[Scratch area at 0 h].

### 2.7. Matrigel Capillary Formation Assay

The Matrigel capillary formation assay was performed as previously described [6,24]. Briefly, EPC were treated as in the cell scratch recovery assay and thereafter trypsinized and resuspended. Growth factor-reduced Matrigel (BD Biosciences) was thawed in an ice bath overnight, and then 10 μL Matrigel was added to a precooled μ-slide (ibidi, Grafelfing, Germany) and incubated at 37 °C for 30 min to polymerize. Thereafter, resuspended EPC (4 × 10^4^ cells per well) from different treatment groups were seeded onto the Matrigel, and the capillaries were recorded 8 h later. The length of the capillaries in the images was measured using ImageJ.

### 2.8. Oxidative Stress Determination

The reactive oxygen species (ROS) content in EPC was determined using Reactive Oxygen Species Assay Kit (Beyotime, Shanghai, China). Briefly, EPC were plated on 24-well plates and treated as in the cell scratch recovery assay. Thereafter, the culture medium was changed to serum-free culture medium containing 10 μM 2,7-Dichlorodi-hydrofluorescein diacetate (DCFH-DA) at 37 °C for 20 min. After entering the cell, DCFH-DA can be hydrolyzed by esterase to produce DCFH, which was then oxidized to fluorescent DCF by intracellular ROS. Cells were washed with serum-free culture medium three times, and fluorescent images (ex/em = 488/525 nm) were captured. The fluorescence intensity was detected using a SynergyH1 microplate reader (Biotek, Burlington, VT, USA).

### 2.9. RNA Sequencing

Total RNA was isolated from EPC treated with HG (*n* = 3) or HG along with 12 μg/mL liraglutide (HG + Lira) using a total RNA isolation kit (Tiangen, Beijing, China), and sent to Novogene (Beijing, China) for RNA sequencing. Briefly, total RNA concentration was quantified using Qubit RNA Assay Kit in Qubit2.0 Flurometer (Life Technologies, Carlsbad, CA, USA), and the integrity of RNA was assessed using the RNA Nano 6000 Assay Kit of the Bioanalyzer 2100 system (Agilent Technologies, Santa Clara, CA, USA). Then, sequencing libraries were generated using NEBNext Ultra RNA Library Prep Kit for Illumina (NEB, Ipswich, MA, USA), and index codes were added to attribute sequences to each sample. The clustering of the index-coded samples was carried out on a cBot Cluster Generation System using TruSeq PE Cluster Kit v3-cBot-HS (Illumia). Thereafter, the library preparations were sequenced on an Illumina Hiseq platform. Raw data were processed through in-house Perl scripts. Differential expression analysis was performed using the DESeq2 R package (1.16.1). Genes with an adjusted *p*-value of < 0.05 found by DESeq2 were assigned as differentially expressed. A volcano plot was used to indicate the difference of gene expression, and a heat map was used to list the differentially expressed genes that we were interested in. Gene Ontology (GO) enrichment analysis of differentially expressed genes was implemented by the clusterProfiler R package.

### 2.10. Detection of Nitric Oxide (NO) Production

NO production was determined as previously described [25]. In brief, EPC were seeded on 12-well plates (2 × 105 cells/well) and maintained under basal culture conditions or with HG (33 mmol/L) for 24 h in the presence or absence of liraglutide. Then, the culture medium in each well was changed to serum-free MCDB131 and cultured for 4 additional hours at 37 °C. Then, the supernatants were collected and NO concentration was determined using a Nitric Oxide Colorimetric Assay Kit (4A biotech Co Ltd., Beijing, China) according to the manufacturer’s protocol. 

### 2.11. RNA Isolation and Semi-Quantitative RT-PCR (sqRT-PCR) 

Total RNA was extracted from EPC using an RNA extraction kit (Tiangen), and then 1μg RNA was reverse-transcribed to cDNA using a high-capacity cDNA reverse transcription kit (Invitrogen). sqRT-PCR was performed using a SYBR Green PCR Master Mix kit (Invitrogen) according to the manufacturer’s instructions on a 7500 Real-Time PCR machine (Applied Biosystems, Carlsbad, CA, USA). The specific primers for human nuclear factor erythroid 2-related factor 2 (Nrf2), Heme Oxygenase 1 (HO-1), and NAD(P)H Quinone Dehydrogenase 1(NQO-1) were purchased from GenScript (Nanjing, China). β-Actin was used as an internal loading control. Gene-specific primer sequences used for sqRT-PCR are listed as follows: 

Nrf2 Sense: 5′-TTCAGCCAGCCCAGCACATC-3′

Antisense: 5′-ACGGGAATGTCTGCGCCAAA-3′

HO1 Sense: 5′-CCAAGGAGGTGCACACCCAG-3′

Antisense: 5′-TGGAGCCGCTTCACATAGCG-3′

NQO1 Sense: 5′-GGTGGTGGAGTCGGACCTCT-3′

Antisense: 5′-CACAAGGTCTGCGGCTTCCA-3′

β-Actin Sense: 5′-GAGCTACGAGCTGCCTGACG-3′

Antisense: 5′-TGCCAGGGCAGTGATCTCCT-3′

### 2.12. Nrf2 Activity Assessment

The activity of Nrf2 was evaluated using ARE firefly luciferase reporter plasmid pARE-luc (D2112, Beyotime, Shanghai, China). EPC were seeded in 48-well culture plates and transfected with pARE-luc and housekeeping plasmid pRL-TK (D2760, Beyotime) using Lipofectamine™ 3000 (Invitrogen). Thereafter, plasmid transfected EPC were treated as in the cell scratch recovery assay. Firefly and Renilla luciferase activities were measured using a Dual-Lumi Luciferase Reporter Gene Assay Kit (RG088, Beyotime), according to the manufacturer’s protocol.

### 2.13. RNA Interference

To knockdown nuclear factor erythroid 2-related factor 2 (Nrf2) expression in EPC, small interfering RNAs (siRNAs) against human Nrf2 purchased from Genepharma (Shanghai, China) were transfected into EPC using Lipofectamine 3000 (Thermo Fisher Scientific, Waltham, MA, USA) according to the manufacturer’s instructions. A total of 72 h after transfection, the expression of Nrf2 and its downstream genes NQO-1 and HO-1 were determined using sqRT-PCR and Western blotting. SiRNAs used for Nrf2 knockdown are listed as follows:

Nrf2-772, sense: GGUUGAGACUACCAUGGUUTT, 

antisense: AACCAUGGUAGUCUCAACCTT; 

Nrf2-811, sense: GACAGAAGUUGACAAUUAUTT, 

antisense: AUAAUUGUCAACUUCUGUCTT; 

Nrf2-1220, sense: CCAGAACACUCAGUGGAAUTT, 

antisense: AUUCCACUGAGUGUUCUGGTT.

### 2.14. Western Blot

Western blot was performed as described in a previous study. Gastrocnemius muscle tissues and harvested cells were homogenized or lysed in ice-cold RIPA lysis buffer (Cell Signaling Technology). The protein concentration was determined using a Bradford protein assay kit (Bio-Rad, Hercules, CA, USA). Equivalent protein concentrations were resuspended in a loading buffer, and then loaded onto a 10% sodium dodecyl sulfate polyacrylamide gel (SDS-PAGE), and electrophoretically separated. Thereafter, proteins were transferred to a polyvinylidene difluoride membrane. After blocking in Tris-buffered saline with Tween (TBST) containing 5% nonfat milk and 0.5% bovine serum albumin for 1 h, membranes were incubated with primary antibodies against CD31, glyceraldehyde-3-phosphate dehydrogenase (GAPDH), phosphorylated endothelial nitric oxide synthase (p-eNOS), eNOS, phosphorylated protein kinase B (p-AKT), AKT, phosphorylated glycogen synthesis kinase-3 beta (p-GSK3β), GSK3β, 4-Hydroxynonenal (4-HNE), β-Actin, NQO-1, HO-1, Nrf2, or tyrosine kinase Fyn (Fyn) overnight at 4 °C. After three washes with TBST, the membranes were incubated with corresponding horseradish peroxidase (HRP)-conjugated secondary antibodies (Cell Signaling Technology) at room temperature for 1 h. After an additional three washes with TBST, blots were visualized with ECL (Thermo Scientific, Waltham, MA, USA) and detected using a Western blot imaging system (Tanon, Shanghai, China).

### 2.15. Statistical Analysis

The results were obtained from at least three independent experiments and are presented as the means ± standard deviations (S.D.). Statistically significant differences between groups were determined with GraphPad Prism 8 software (GraphPad) using one-way ANOVA and Student’s *t*-test where appropriate. Statistical significance was defined as a *p*-value less than 0.05.

## 3. Results

### 3.1. Liraglutide Improves Blood Perfusion and Angiogenesis in Ischemic Hindlimb of T2DM Mice

**Figure 1:**
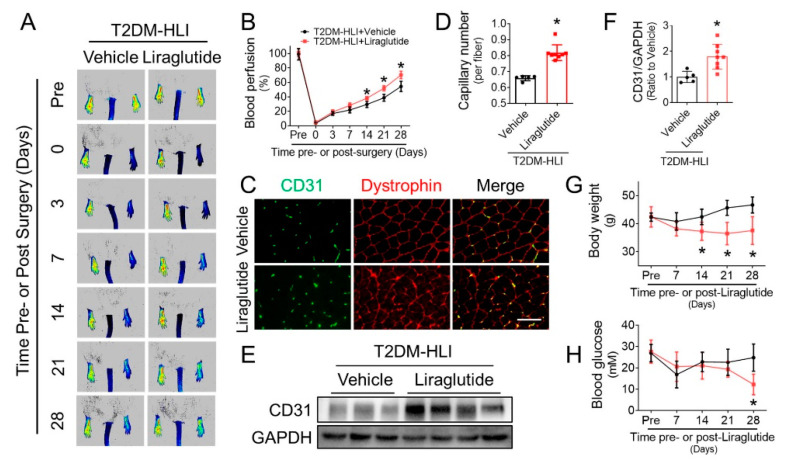
Liraglutide improves blood perfusion and angiogenesis in ischemic hindlimbs of T2DM mice. The proangiogenic effect of liraglutide in diabetic ischemic tissues was investigated in db/db mice. The blood perfusion in ischemic hindlimb of db/db mice treated with or without liraglutide was evaluated using a PeriCam PSI (**A**), and quantified using Image J (**B**). At the 28th day after HLI surgery, CD31-positive capillaries in gastrocnemius muscle tissue of ischemic hind limbs were detected by immunofluorescent staining (**C**), and presented as CD31-positive capillaries per muscle fiber identified by dystrophin staining (**D**). CD31 expression in ischemic hind limb was also detected by Western blot (**E**) and quantified using Imagequant (**F**), glyceraldehyde 3-phosphate dehydrogenase (GAPDH) was used as loading control. Blood glucose (**G**) and body weight (**H**) were monitored before and after HLI surgery. *n* = 5 or 8 mice per group. Data shown in graphs presented as mean ± S.D. * *p* < 0.05 vs vehicle group, Bar = 100 μm. T2DM, type 2 diabetes mellitus.

Blood perfusion in the ischemic hindlimb of db/db mice was evaluated by PSI. Results showed that blood perfusion in the ischemic hindlimbs of the liraglutide-treated mice obviously exceeded that of the vehicle group mice following the 14th day post-surgery (Figure 1A,B). To evaluate whether liraglutide promotes angiogenesis, capillaries in ischemic gastrocnemius muscle were indicated by CD31 staining. Immunofluorescence staining showed there were more capillaries in the ischemic gastrocnemius of the liraglutide-treated mice than that of the vehicle group mice (Figure 1C,D). In addition, western blot also showed that CD31 content in the ischemic gastrocnemius lysate of the liraglutide-treated mice was obviously higher than that of the vehicle group mice (Figure 1E,F). These results demonstrated that liraglutide improves angiogenesis and enhances blood perfusion in the ischemic tissues of T2DM-HLI mice.

As a widely used medicine in the treatment of T2DM, liraglutide has a well-established antihyperglycemic effect [14]. It lowered blood glucose levels (Figure 1G) and reduced body weight (Figure 1H) in T2DM mice. However, the beneficial effects of liraglutide on enhancing blood perfusion appeared earlier than its effects on lowering blood glucose levels (14th day vs. 28th post-surgery, respectively), which implies that the capability of liraglutide in improving ischemic angiogenesis and promoting blood perfusion under diabetic conditions may be independent of its effects on lowering blood glucose.

### 3.2. Liraglutide Improves Blood Perfusion and Angiogenesis in Ischemic Hindlimb of T1DM Mice

**Figure 2:**
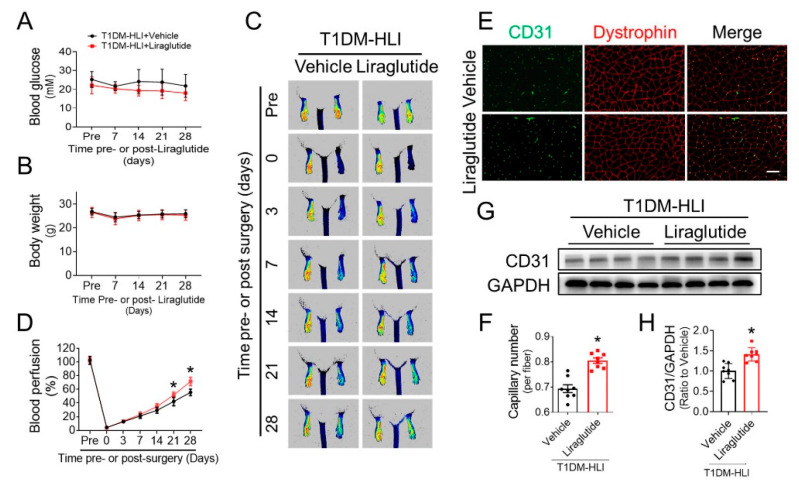
Liraglutide improves blood perfusion and angiogenesis in ischemic hindlimbs of T1DM mice. The proangiogenic effect of liraglutide in ischemic tissue was also investigated in STZ-induced T1DM mice. Blood glucose (**A**) and body weight (**B**) were monitored before and after HLI surgery. Blood perfusion in ischemic hindlimb of T1DM mice was evaluated using a PeriCam PSI (**C**), and quantified using Image J (**D**). At the 28th day post HLI surgery, capillaries in gastrocnemius muscle tissue of ischemic hind limbs were detected by CD31 staining (**E**), and presented as CD31-positive capillaries per muscle fiber (**F**). CD31 expression in ischemic gastrocnemius muscle was also detected by Western blot (**G**) and quantified using Imagequant (**H**), and GAPDH was used as loading control. Data shown in graphs presented as mean ± S.D. *n* = 8 mice per group. * *p* < 0.05 vs Vehicle group. Bar = 100 μm. T1DM, type 1 diabetes mellitus.

To further confirm whether the beneficial effects of liraglutide on promoting blood perfusion and angiogenesis under diabetic conditions depend on its effects on lowering blood glucose levels and/or reducing body weight, we evaluated the effect of liraglutide in improving blood perfusion and angiogenesis using T1DM mice with HLI. Liraglutide did not affect the blood glucose level or body weight of T1DM mice (Figure 2A,B). PSI results showed that blood perfusion recovery in ischemic hindlimbs of the liraglutide-treated group was superior to that of the vehicle group following the 21st day post-surgery (Figure 2C,D), indicating that liraglutide obviously improves blood perfusion under T1DM conditions. Moreover, CD31 staining showed a higher capillary density in ischemic gastrocnemius muscle of the liraglutide-treated mice than that of the vehicle-treated mice (Figure 2E,F), and CD31 content in the ischemic gastrocnemius lysate of the liraglutide-treated mice was obviously higher than that of the vehicle group mice (Figure 2G,H). Thus, liraglutide promotes diabetic ischemic angiogenesis, at least partially, independent of its effects on lowering blood glucose and/or body weight.

### 3.3. Liraglutide Ameliorates the Function of HG-Treated EPC

**Figure 3:**
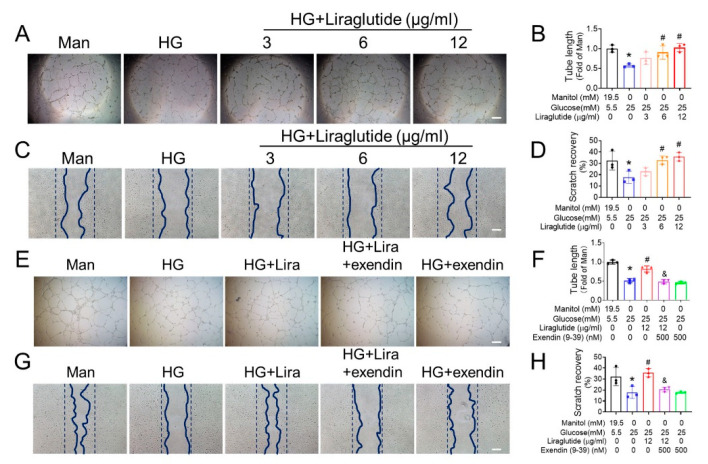
Liraglutide improves angiogenic function of HG-treated EPC. EPC were treated with HG at the presence or absence of liraglutide for 24 h, and mannitol (Man) was used as an osmotic control. The dosage effect of liraglutide on the angiogenic function of HG-treated EPC were evaluated by Matrigel tube formation assay (**A**), and the tube length was quantified using Image J and normalized to the Man treatment group (**B**). The effects of liraglutide on EPC migration were evaluated by cell scratch assay (**C**), and the scratch healing was quantified using Image J and normalized to its initial scratch area (**D**). The effect of GLP-1R antagonist exendin (9–39) on liraglutide enhancing angiogenic function of HG-treated EPC was also evaluated by Matrigel tube formation assay (**E**,**F**) and cell scratch assay (**G**,**H**). Three independent experiments were performed for each study. Data shown in graphs represent as mean ± S.D. * *p* < 0.05 vs. Man group, # *p* < 0.05 vs. HG treatment group, and & *p* < 0.05 vs. HG + liraglutide group. Bar = 100 μm.

Considering the pivotal role of EPC dysfunction in causing the impairment of angiogenesis under diabetic conditions, it was investigated whether liraglutide improves EPC dysfunction under diabetic conditions. EPC (CD144+/CD34+/VEGFR2+/CD14−/CD45−, Figure S1) were exposed to HG to mimic the hyperglycemia under diabetic conditions. Liraglutide has a high safe dose for EPC (Figure S2), and the Matrigel tube formation assay showed that the tube formation capability of EPC was significantly impaired under HG conditions, while restored by liraglutide in a dose-dependent manner (Figure 3A,B). The scratch recovery assay presented similar results. EPC migration was inhibited under HG conditions, while liraglutide treatment improved the migration of HG-treated EPC in a dose-dependent manner (Figure 3C,D). Pretreating EPC with exendin 9–39 (exendin), a well-established antagonist of GLP-1R, abolished the protective effect of liraglutide against HG-induced impairment on tube formation (Figure 3E,F) and scratch recovery (Figure 3G,H), indicating that liraglutide improves HG-treated EPC function via its receptor GLP-1R instead of its own biochemical properties.

### 3.4. Liraglutide Attenuates HG-Induced Oxidative Stress in EPC

**Figure 4:**
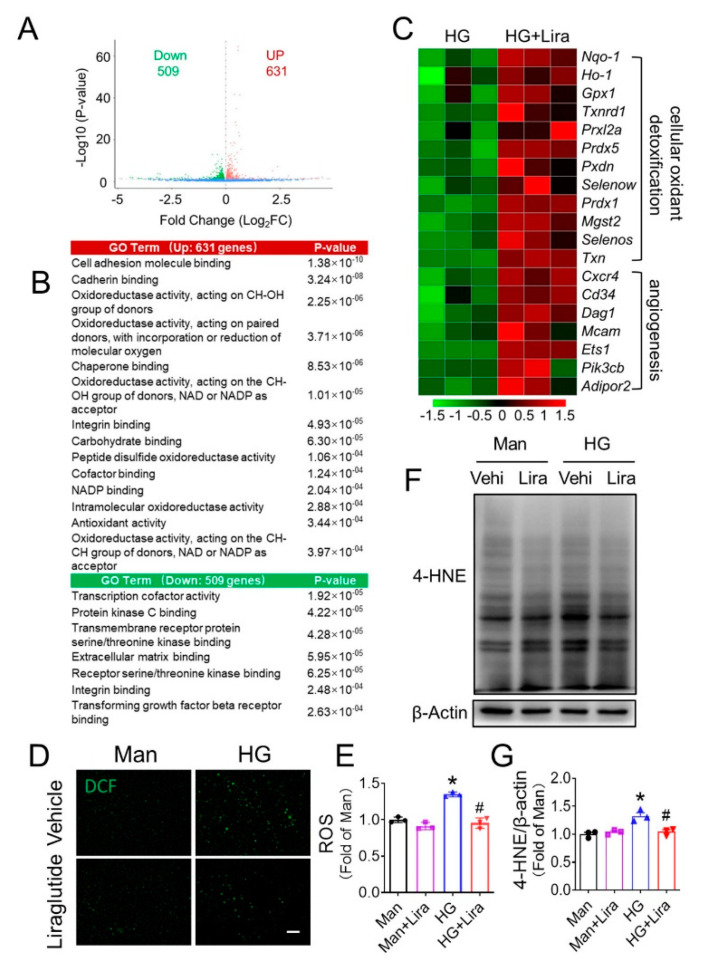
Liraglutide ameliorates oxidative stress in HG-treated EPC. EPC were treated with HG or HG plus liraglutide (HG + Lira) for 24 h, and total RNA was isolated and analyzed by RNAseq (*n* = 3 per group). Genome-wide changes in mRNA expression shown in a volcano plot, and the numbers refer to the number of genes up- or down-regulated by two-fold or more with a *p* value < 0.05 (**A**). GO enrichment analysis indicates the biological pathways and molecular functions that is significantly different between HG + Lira group and HG group (**B**). The selected genes with differential expression between HG and HG + Lira group are listed in heatmap (**C**). Oxidative stress in EPC treated with Man or HG in the present or absent of liraglutide were indicated by DCF staining (**D**,**E**), and oxidative stress marker 4-HNE was detected by Western blot (**F**,**G**). Data shown in graphs represent as mean ± S.D. * *p* < 0.05 vs. Man group; # *p* < 0.05 vs. HG group. Bar = 100 μm.

RNA sequencing was performed to compare the mRNA expression patterns of HG-treated EPC in the presence or absence of liraglutide, to explore the underlying mechanism how liraglutide improves the function of HG-treated EPC. Results showed that 631 genes were upregulated, and 509 genes were downregulated by liraglutide (Figure 4A). For the GO analyses, GO terms related with angiogenesis and antioxidative activity were highly overrepresented in the HG plus liraglutide group (HG + Lira) compared with the HG plus vehicle group (HG) (Figure 4B). Indeed, overexpressed genes in the HG + Lira group included those related to antioxidative activity and angiogenesis (Figure 4C), which is consistent with the beneficial effect of liraglutide in promoting angiogenic function of HG-treated EPC (Figure 3). DCF staining showed that ROS production was increased in HG-treated EPC and significantly alleviated by liraglutide (Figure 4D,E), which was consistent with the expression of 4-HNE, one of the major end products of lipid oxidation (Figure 4F,G).

NO plays a critical role in maintaining the function of EPC and promoting ischemic angiogenesis [26]. Impairment in NO bioavailability induced by oxidative stress is one major cause of EPC dysfunction under diabetic conditions [27]. We found that HG treatment significantly decreased the NO contents in the supernatant and cell lysate of EPC, while liraglutide can obviously reverse the impaired NO bioavailability (Figure S3A,B). Similarly, the phosphorylation of eNOS at Ser1177, which determines eNOS activity and NO production, was also repressed in HG-treated EPC and restored after liraglutide treatment (Figure S3C,D). These results indicated that repressing oxidative stress may be a critical component of the mechanism by which liraglutide promotes the angiogenic function of HG-treated EPC. 

### 3.5. Liraglutide Enhances Nrf2 Activity in HG-Treated EPC 

**Figure 5:**
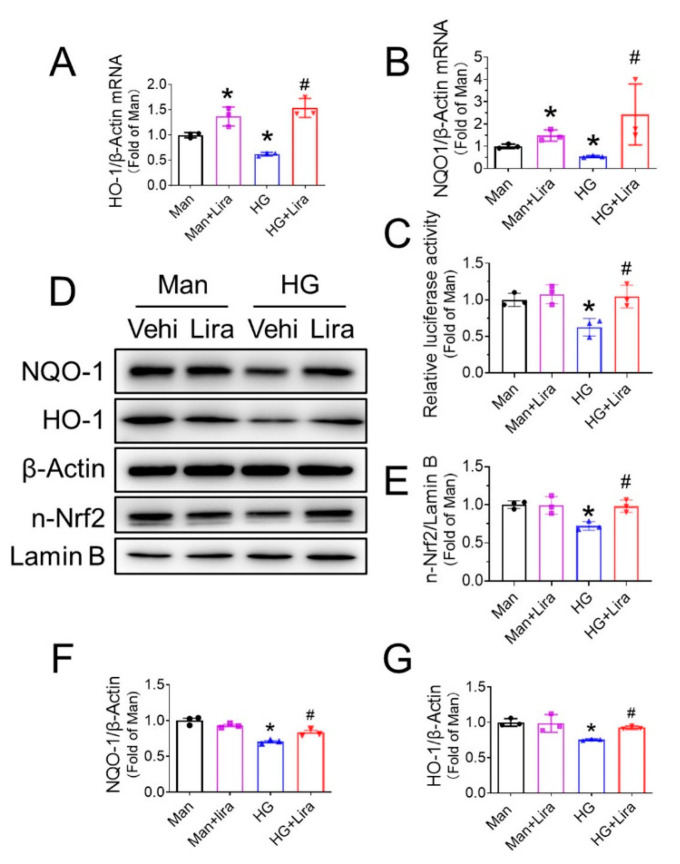
Liraglutide activates Nrf2 in HG-treated EPC. The mRNA expression of HO-1 (**A**) and NQO-1 (**B**) in EPC from different groups was detected by sqRT-PCR. The activity of Nrf2 was evaluated by dual-luciferase assay using ARE luciferase reporter plasmid (**C**). The expression of HO-1, NQO-1 in whole cell lysis and the expression of Nrf2 in nuclear were determined by western blot (**D**), and quantified using Imagequant (**E**–**G**). β-Actin and Lamin B were used as loading control of whole cell lysate and nuclear lysate, respectively. Three independent experiments were performed for each study. * *p* < 0.05 vs. Man group; # *p* < 0.05 vs. HG group.

RNA sequencing results revealed liraglutide increased the expression NQO-1 and HO-1, typical downstream genes of Nrf2 [4,28], in HG-treated EPC (Figure 4C), and the upregulation of mRNA levels of NQO-1 and HO-1 was confirmed by RT-PCR (Figure 5A,B). We then investigated whether liraglutide can activate Nrf2 via examining the impact of liraglutide on ARE-driven luciferase activity. As shown, ARE luciferase activity in EPC was decreased under HG conditions, while increased by liraglutide (Figure 5C). Western blot results also showed that the nuclear Nrf2 content (Figure 5D,E), as well as the protein expression of its downstream genes NQO-1 and HO-1 (Figure 5D,F,G), were significantly decreased in HG-treated EPC but obviously increased after liraglutide administration. These results confirmed that liraglutide enhanced Nrf2 activity in HG-treated EPC.

### 3.6. Nrf2 Mediates the Beneficial Effects of Liraglutide on HG-Treated EPC

**Figure 6:**
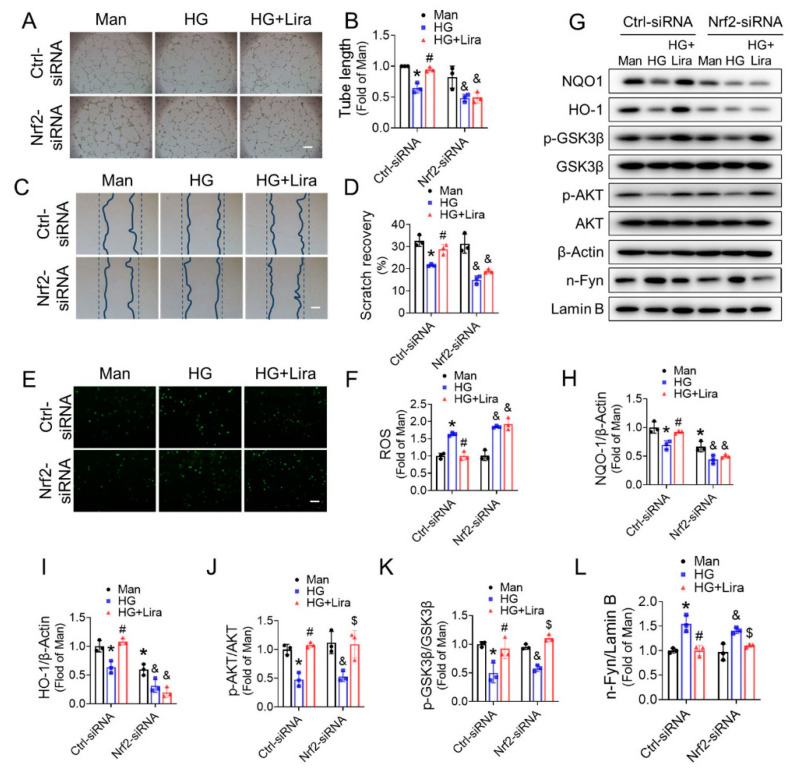
Nrf2 mediates the protective effects of liraglutide against HG-induced EPC dysfunction. The effect of Nrf2-siRNA transfection on the angiogenic function, cell migration and oxidative stress of EPC treated with Man, HG or HG + Lira were investigated by Matrigel tube formation assay (**A**,**B**), cell scratch assay (**C**,**D**), and DCF staining (**E**,**F**), respectively. The effects of Nrf2 knockdown on the expression of NQO-1 (**G**,**H**), HO-1 (**G**,**I**), the phosphorylation of AKT (**G**,**J**), GSK3β (**G**,**K**), nuclear content of Fyn (**G**,**L**) in EPC were determined by western blot and quantified using Imagequant. Three independent experiments were performed for each study. Data shown in graphs represent the mean ± S.D. * *p* < 0.05 vs. Man group, # *p* < 0.05 vs. Ctrl-siRNA HG treatment group, & *p* < 0.05 vs. Nrf2-siRNA + Man group, $ *p* < 0.05 vs. Nrf2-siRNA + HG group. Bar = 100 μm.

To confirm the crucial role of Nrf2 in liraglutide ameliorating diabetic EPC function, Nrf2 expression was knocked down by Nrf2 siRNA. Notably, siRNA811 had the best knockdown efficiency (Figure S4A,B) among the three Nrf2-siRNAs tested, and this siRNA was used to knockdown Nrf2 in subsequent studies (Figure S4C,D). Knockdown of Nrf2 abolished most of the beneficial effects of liraglutide, including tube formation (Figure 6A,B) and cell migration (Figure 6C,D), on HG-treated EPC. Moreover, Nrf2 knockdown also eliminated the protective effect of liraglutide against oxidative stress in HG-treated EPC (Figure 6E,F) and the upregulation of NQO-1 (Figure 6G,H) and HO-1 (Figure 6G,I). Moreover, the effect of liraglutide in increasing NO content (Figure S5A) and promoting eNOS phosphorylation (Figure S5B,C) in HG-treated EPC were abolished after Nrf2 knockdown. These results suggest that Nrf2 mediates the protective effects of liraglutide on HG-treated EPC.

### 3.7. Nrf2 Deletion Attenuates the Beneficial Effect of Liraglutide in Improving Blood Perfusion and Angiogenesis in Ischemic Hindlimb of T1DM Mice

**Figure 7:**
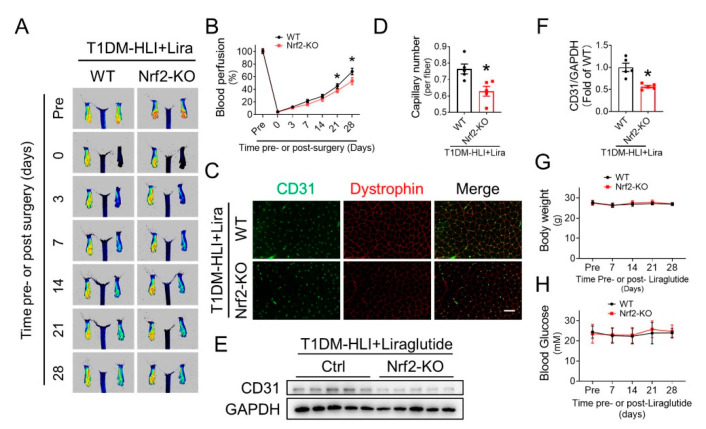
Nrf2 deficiency attenuates the beneficial effect of liraglutide in improving blood perfusion and angiogenesis in ischemic hindlimbs of T1DM mice. The proangiogenic effect of liraglutide in ischemic tissue was investigated in WT-T1DM and Nrf2-KO-T1DM mice. Blood perfusion in ischemic hindlimb was evaluated using a PeriCam PSI (**A**), and quantified using Image J (**B**). At the 28th day after HLI surgery, capillaries in gastrocnemius muscle tissue of ischemic hind limbs were detected by CD31 staining (**C**), and presented as CD31-positive capillaries per muscle fiber (**D**). CD31 content in ischemic gastrocnemius muscle was also detected by Western blot (**E**) and quantified using Imagequant (**F**), and GAPDH was used as loading control. Blood glucose (**G**) and body weight (**H**) were monitored before and after HLI surgery. Data shown in graphs presented as mean ± S.D. *n* = 5 mice per group. * *p* < 0.05 vs. T1DM-HLI WT mice. Bar = 100 μm. T1DM, type 1 diabetes mellitus.

To further confirm the importance of Nrf2 in mediating the beneficial effect of liraglutide, we evaluated whether Nrf2 deficiency affects the effect of liraglutide on improving blood perfusion and angiogenesis under diabetic conditions. Both Nrf2-KO mice and wild type (WT) mice were used to establish a T1DM-HLI model and received liraglutide administration. PSI results showed that the liraglutide-induced blood perfusion recovery in ischemic hindlimbs was attenuated in Nrf2-KO mice following the 21st day post-surgery (Figure 7A,B), indicating that the beneficial effect of liraglutide in promoting blood perfusion relies on Nrf2. Moreover, CD31 staining showed a lower capillary density in the ischemic gastrocnemius muscle of Nrf2-KO mice (Figure 7C,D), as well as less CD31 content in the ischemic gastrocnemius lysate (Figure 7E,F), than that of WT mice. Furthermore, the blood glucose level and body weight of WT or Nrf2-KO T1DM mice were not affected by liraglutide (Figure 7G,H). These results demonstrated that the beneficial effects of liraglutide in promoting diabetic ischemic angiogenesis rely on Nrf2 activation.

### 3.8. Liraglutide Activates Nrf2 through the AKT/GSK-3β/Fyn Pathway

**Figure 8:**
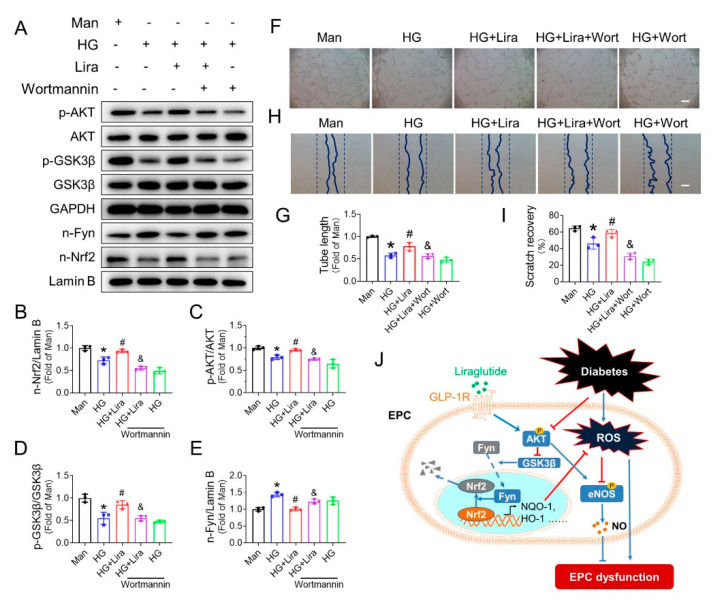
Liraglutide activates Nrf2 via the AKT-GSK3β-Fyn pathway. The nuclear content of Nrf2 (**A**,**B**), the phosphorylation of AKT (**A**,**C**) and GSK3β (**A**,**D**), and nuclear content of Fyn (**A**,**E**) in HG-treated EPC receiving liraglutide treatment in the presence or absence of wortmannin were determined by western blot and quantified using Imagequant. The effect of wortmannin on liraglutide improving angiogenic function of HG-treated EPC was evaluated by Matrigel tube formation assay (**F**) and quantified using Image J (**G**). The effect of wortmannin on liraglutide enhancing migratory function of HG-treated EPC was investigated by cell scratch assay (**H**,**I**). Three independent experiments were performed for each study. * *p* < 0.05 vs. Man group; # *p* < 0.05 vs. HG group, & *p* < 0.05 vs. HG + Lira. Bar = 100 μm. (**J**) Schematic illustration of the protective effects of liraglutide on EPC under diabetic conditions. Diabetes mellitus induces oxidative stress, which impairs the angiogenic function of EPC. Liraglutide improves EPC function predominantly via Nrf2 activation mediated by increased phosphorylation of AKT and GSK-3β and inhibiting Fyn-mediated export and degradation of nuclear Nrf2. NQO-1, NAD(P)H dehydrogenase (quinone1); HO-1, heme oxygenase-1; ROS, reactive oxygen species; eNOS, endothelial nitric oxide synthase; NO, nitric oxide.

To further investigate the underlying mechanism of how liraglutide activates Nrf2, we detected the activity of the AKT/GSK-3β/Fyn pathway, which inhibits Nrf2 activity via enhancing its nuclear export [29]. HG decreased nuclear content of Nrf2 (Figure 8A,B), repressed the phosphorylation of AKT (Figure 8A,C) and GSK3β (Figure 8A,D), while it increased nuclear Fyn content (Figure 8A,E) in EPC, which was substantially reversed after liraglutide treatment. In addition, GLP-1R antagonist exendin eliminated the beneficial effects of liraglutide on activating the AKT pathway (Figure S6), including elevated phosphorylation of AKT (Figure S6A,B) and GSK3β (Figure S6A,C), reduced nuclear Fyn content (Figure S6A,D) and increased nuclear accumulation of Nrf2 (Figure S6A,E), which further confirmed that liraglutide activates the AKT/GSK-3β/Fyn pathway.

Wortmannin, a specific inhibitor of AKT, abolished the effect of liraglutide on activating the AKT/GSK-3β/Fyn pathway and promoting Nrf2 activation (Figure 8A–E). On the other hand, knockdown Nrf2 with siRNA did not affect the activation of the AKT/GSK-3β/Fyn pathway induced by liraglutide (Figure 6G,J–L). These factors demonstrated that liraglutide restores Nrf2 activity in HG-treated EPC via AKT pathway. Additionally, the beneficial effect of liraglutide in improving NO content (Figure S7A), as well as the phosphorylation of eNOS (Figure S7B,C) were also abrogated by wortmannin. Moreover, wortmannin completely abolished the effect of liraglutide on promoting tube formation (Figure 8F,G) and cell migration (Figure 8H,I) of HG-treated EPC, which further confirmed the critical role of the AKT pathway in mediating the beneficial effects of liraglutide. 

## 4. Discussion

EPC dysfunction in patients with T1DM [12] or T2DM [30] is considered as one major cause of the development and progression of diabetic vascular complications. Thus, improving EPC function is considered as a promising strategy for the treatment of diabetic vascular complications [31]. In the present study, three new lines of evidence reveal the beneficial effects of liraglutide on ischemic angiogenesis in diabetic mice and the angiogenic function of EPC: (1) liraglutide enhances blood perfusion and angiogenesis in the ischemic hindlimb of both T1DM and T2DM mice; (2) liraglutide promotes the angiogenic capability of HG-treated EPC by activating Nrf2, which also restores the eNOS phosphorylation and thereafter NO production of EPC; (3) liraglutide promotes the transcriptional activity of Nrf2 via an AKT-GSK3β-Fyn pathway.

The first important finding in the present study is that liraglutide can effectively promote ischemic angiogenesis under diabetic conditions. Liraglutide aids in glycemic control by repressing appetite, stimulating postprandial release of insulin and reducing glucagon secretion [16], and has become a first-line medication for T2DM [14]. In addition to its antidiabetic activity, recent studies have revealed the protective effect of liraglutide against the comorbidities and complications of T2DM [32]. *The Liraglutide Effect and Action in Diabetes: Evaluation of Cardiovascular Outcome Results* (LEADER, NCT01179048) trial reported that liraglutide leads to significant relative risk reduction in major adverse cardiovascular events (MACE) in T2DM patients with a high cardiovascular risk [17], chronic kidney disease [18], poly vascular or single vascular disease [19], or a history of myocardial infarction or stroke [20], demonstrating that liraglutide can ameliorate cardiovascular complications of T2DM. Consistent with the conclusions of the LEADER trial, we also found that liraglutide promoted blood perfusion and angiogenesis in ischemic tissue under T2DM conditions (Figure 1). However, it remains unknown whether the beneficial effects of liraglutide on diabetic cardiovascular complications completely rely on its blood glucose and body weight lowering capabilities. In the present study, we found that liraglutide alone did not lower the blood glucose and body weight of T1DM mice (Figure 2A,B), but still promoted blood perfusion and angiogenesis in ischemic tissue (Figure 2C–H). Though the blood glucose and body weight lowering effect of liraglutide contributes to its effect of improving ischemic angiogenesis under diabetic conditions, our findings indicate that the pro-angiogenic effects of liraglutide is partially independent of its hypoglycemic and body weight lowering effects. In line with our findings, liraglutide had been demonstrated to protect against the cardiopulmonary complications in T1DM rats [33] and improve the function of the enteric nervous system in T1DM patients [34] without significant effects on their blood glucose levels and body weights. Moreover, liraglutide has also been shown to ameliorate arterial restenosis in a femoral artery wire injury model [35] and attenuate cardiovascular complications of an arterial hypertension model [36] under non-diabetic conditions. These findings, along with our present study, strongly indicate direct vascular protective effects of liraglutide. 

EPC function is impaired in diabetes and improving EPC function is considered as a promising strategy for the treatment of diabetic vascular complications [31]. Therefore, we investigated whether liraglutide directly affects the function of diabetic EPC. We found that liraglutide treatment improved the migration and tube formation of HG-treated EPC in a dose-dependent manner (Figure 3A–D), while GLP-1R antagonist exendin abolished these protective effects (Figure 3E–H), demonstrating that liraglutide directly improves the function of diabetic EPC in addition to its effects of lowering blood glucose and body weight. Besides that, liraglutide may also preserve the angiogenic signals, such as VEGF [37] and subsequently improve the function of diabetic EPC.

Our findings are consistent with the effects of other antidiabetic drugs, including metformin [38], thiazolidinediones [39], statins [40], dipeptidyl peptidase-4 inhibitors [41], and sodium-dependent glucose transporter-2 inhibitor [42], which has been reported to enhance EPC mobilization and/or improve EPC function. Moreover, some existing research [43] has showed that liraglutide can improve the function of diabetic endothelial cells, and the protective effect of liraglutide on the function of resident endothelial cells in ischemic tissue may also contribute to enhanced blood perfusion and angiogenesis. 

Our second innovative finding is that the beneficial effects of liraglutide against EPC dysfunction relies on the activation of Nrf2. The excessive generation of reactive oxygen species (ROS) [44] and consequent impairment in nitric oxide (NO) production [38] are reported to contribute to EPC dysfunction under both type 1 and type 2 diabetes [45], therefore anti-oxidative is considered as effective strategy in promoting EPC function. In the present study, we found that the ROS level in HG-treated EPC was increased, but obviously inhibited by liraglutide treatment (Figure 4D–G and Figure 6E,F), indicating that liraglutide improved the antioxidative capability of EPC, and the phosphorylation of eNOS and the production of NO showed a similar pattern (Figure S3). 

We had previously described that Nrf2 is a critical redox sensor and one of the master regulators of the antioxidative responses [28]. As a transcription factor, Nrf2 binds to regulatory antioxidant response elements and activates transcription of many antioxidative genes, including HO-1 and NQO-1 that counteract ROS. In this study, we demonstrated that liraglutide significantly increased the nuclear content of Nrf2, and therefor upregulated the expression of its downstream antioxidative genes HO-1 and NQO-1 (Figure 4C and Figure 5). These results imply that liraglutide improving EPC antioxidative capacity is likely mediated by the activation of Nrf2. Indeed, the essential role of Nrf2 in maintaining endothelial function has been widely appreciated. Florczyk and colleagues [46] found that a lack of Nrf2 diminished the capillary formation ability of aortic endothelium. Our previous study also showed that Nrf2 knockdown nearly abolished the protective effects of SDF-1/CXCR7 axis against apoptosis and the dysfunction of diabetic EPC [4]. Consistent with these findings, we found that Nrf2 knockdown abolished the protective effects of liraglutide against an elevated ROS level, impaired the cell migration and tube formation capabilities of HG-treated EPC (Figure 6A–F), as well as the activity of eNOS and NO production (Figure S5). Moreover, Nrf2 deficiency attenuated the beneficial effect of liraglutide in improving blood perfusion and angiogenesis in the ischemic hindlimb of T1DM mice (Figure 7). These results confirmed that Nrf2 plays a pivotal role in liraglutide augmenting EPC function. 

The third novel finding is that the AKT/GSK-3β/Fyn pathway mediates liraglutide-induced Nrf2 activation in EPC. The mechanisms of Nrf2 activation have been widely studied under varied pathophysiological conditions. In human umbilical vein endothelial cells, atheroprotective laminar flow activates Nrf2 via the PI3K/AKT pathway [47]. In hippocampal neurons, flurbiprofen triggers the phosphorylation of AKT and GSK3β, which in turn decreases Nrf2 nuclear export and increases Nrf2 translocation [48]. In our previous study, AKT/GSK3β also mediates SDF-1/CXCR7 axis, activating Nrf2 activity [4]. It is also known that Fyn can phosphorylate Nrf2 tyrosines, promoting Nrf2 nuclear export and degradation [49]. Moreover, it was reported that liraglutide activates PI3K/AKT in cardiac microvascular endothelial cells [50] and vascular smooth muscle cells [51]. In the present study, we found that liraglutide increased the phosphorylation of AKT and GSK-3β, which was accompanied by a decreased nuclear content of Fyn and increased nuclear accumulation of Nrf2 (Figure 6G–L and Figure 8A–E). Importantly, we found that a PI3K/AKT inhibitor wortmannin almost completely abolished the activation of Nrf2 induced by liraglutide, as well as its beneficial effects on the migration and tube formation capability of HG-treated EPC (Figure 8F–I), demonstrating that liraglutide activates Nrf2 via the AKT/GSK-3β/Fyn pathway. 

In summary, liraglutide enhances ischemic angiogenesis under diabetic conditions, partially through a mechanism independent of its antihyperglycemic effects. Liraglutide improves EPC dysfunction under diabetic conditions predominantly through increasing Nrf2 activation via the AKT/GSK-3β/Fyn pathway, and subsequently increases antioxidative genes expression, ameliorates oxidative stress and restores the bioavailability of NO, therefore protecting EPC from diabetes and HG-induced dysfunction, and promoting ischemic angiogenesis under diabetic conditions, as illustrated in Figure 8J.

## Data Availability

The data presented in this study are available on request from the corresponding author.

## Supplementary Materials

The following supporting information can be downloaded at: https://www.mdpi.com/article/10.3390/cells11233821/s1, Figure S1: EPC identification; Figure S2: Determining the safe dose of liraglutide in treating EPC; Figure S3: Liraglutide restored NO bioavailability in HG-treated EPC; Figure S4: The knockdown efficiency of siRNA against Nrf2; Figure S5: Nrf2 Knockdown abolished the effect of liraglutide in restoring NO bioavailability in HG-treated EPC; Figure S6: Exendin eliminated the effects of liraglutide on AKT pathway activation in HG-treated EPC; Figure S7: Wortmannin abrogated the effect of liraglutide in restoring NO bioavailability in HG-treated EPC.
