# Supplementary material for: Liraglutide Improves the Angiogenic Capability of EPC and Promotes Ischemic Angiogenesis in Mice under Diabetic Conditions through an Nrf2-Dependent Mechanism
Source: Cells. 2022 Nov 29;11(23):3821. doi: 10.3390/cells11233821 (PMC9736458; doi:10.3390/cells11233821)
Supplement: Supplementary file 1 [file cells-11-03821-s001.zip › cells-1918638-supplementary.pdf]

Supplementary Figures

Figure S1

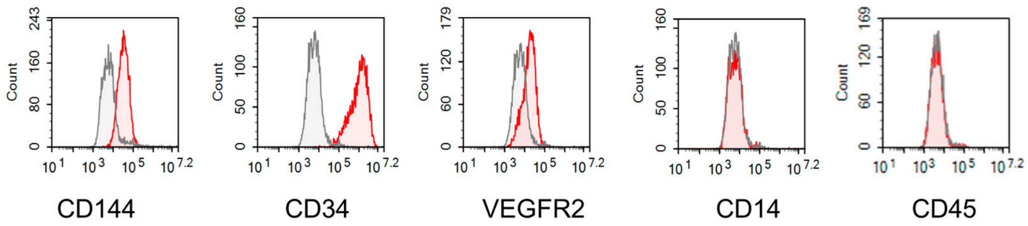

Figure S1. EPCs identification. EPCs were identified by detecting cell surface biomarkers via flow cytometry. EPCs were positive for CD144, CD34 and VEGFR2, and negative for CD14 and CD45.

Figure S2

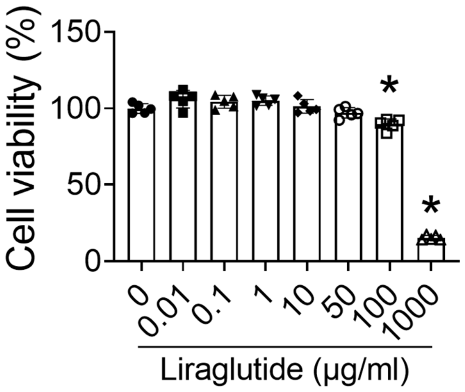

Figure S2. Determining the safe dose of liraglutide in treating EPCs. EPCs were treated with liraglutide at the dosage ranged from 0.01, 0.1, 1, 10, 50, 100 and 1000 µg/ml for 48h, and cell viability was evaluated by CCK-8 kit. The dose of liraglutide that did not affect the cell viability was considered as safe dosage. * $p<0.05$ vs. 0 µg/ml liraglutide.

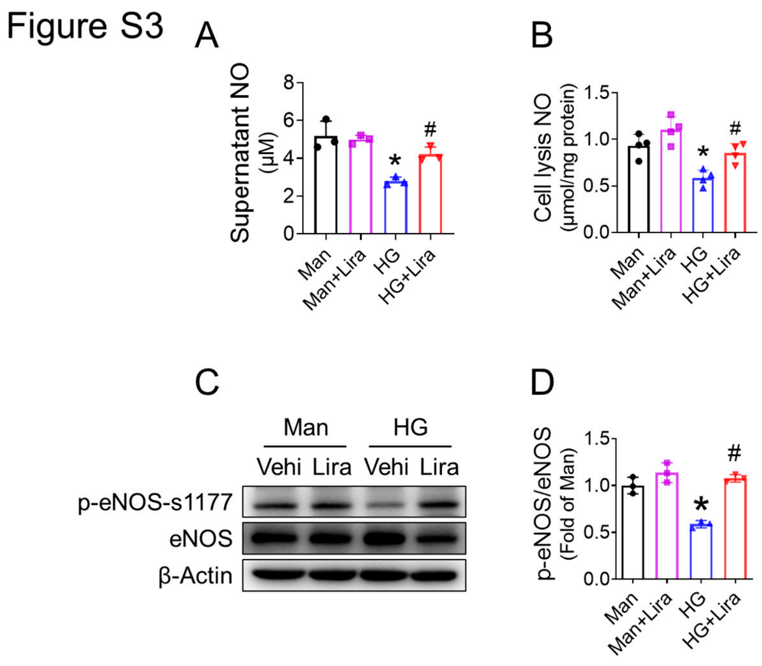

Figure S3. Liraglutide restored NO bioavailability in HG-treated EPCs. NO content in the supernatant (A) and cell lysis (B) of EPCs from different groups were detected using NO Assay kit. The phosphorylation of eNOS at Ser1177 was detected by Western blot (C) and quantified using Imagequant (D). Data shown in graphs represent the mean ± S.D. n = 3 or 4 per group. β-Actin was used as loading control. *P < 0.05 vs. Mannitol group; #P < 0.05 vs. HG group.

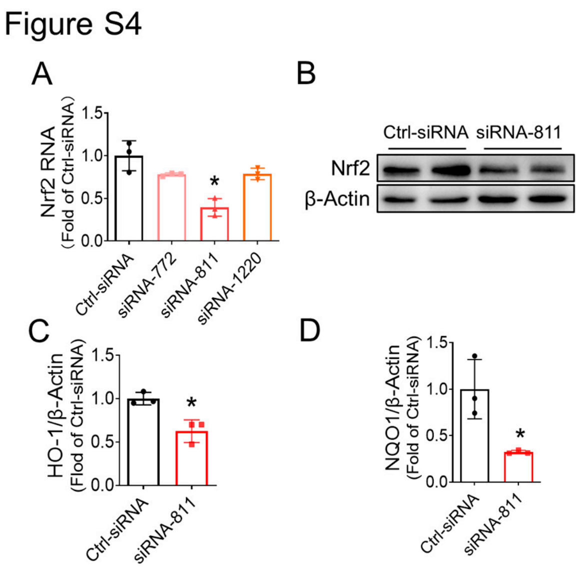

Figure S4. The knockdown efficiency of siRNA against Nrf2. The knockdown efficiency of siRNA against Nrf2 was determined by sqRT-PCR (A) and Western blot

(B). The effects of Nrf2-siRNA on Nrf2 activity were determined by HO-1(C) and NQO-1 (D) expression via sqRT-PCR. Data shown in graphs represent the mean ± S.D. n = 3 per group. *P < 0.05 vs. Ctrl-siRNA group.

Figure S5

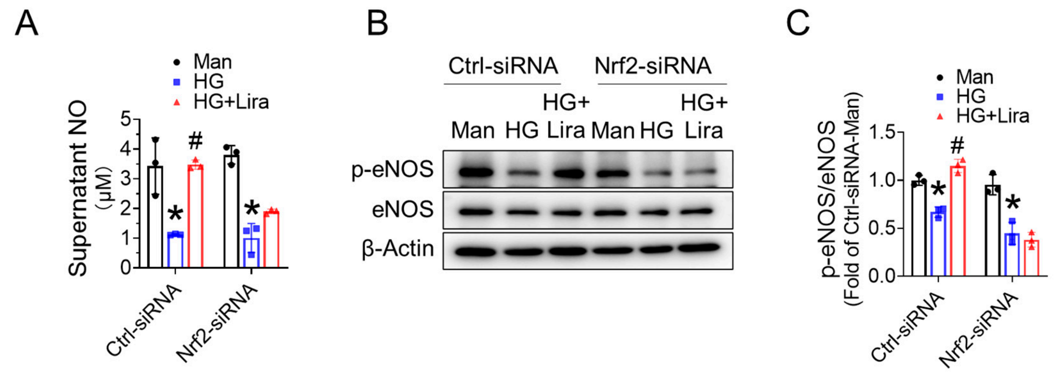

Figure S5. Nrf2 Knockdown abolished the effect of liraglutide in restoring NO bioavailability in HG-treated EPCs. NO content in the supernatant (A) of EPCs from different groups were detected using NO Assay kit. The phosphorylation of eNOS at Ser1177 was detected by Western blot (B) and quantified using Imagequant (C). Data shown in graphs represent the mean ± S.D. n = 3 per group. β-Actin was used as loading control. *P < 0.05 vs. Mannitol group; #P < 0.05 vs. HG group.

Figure S6

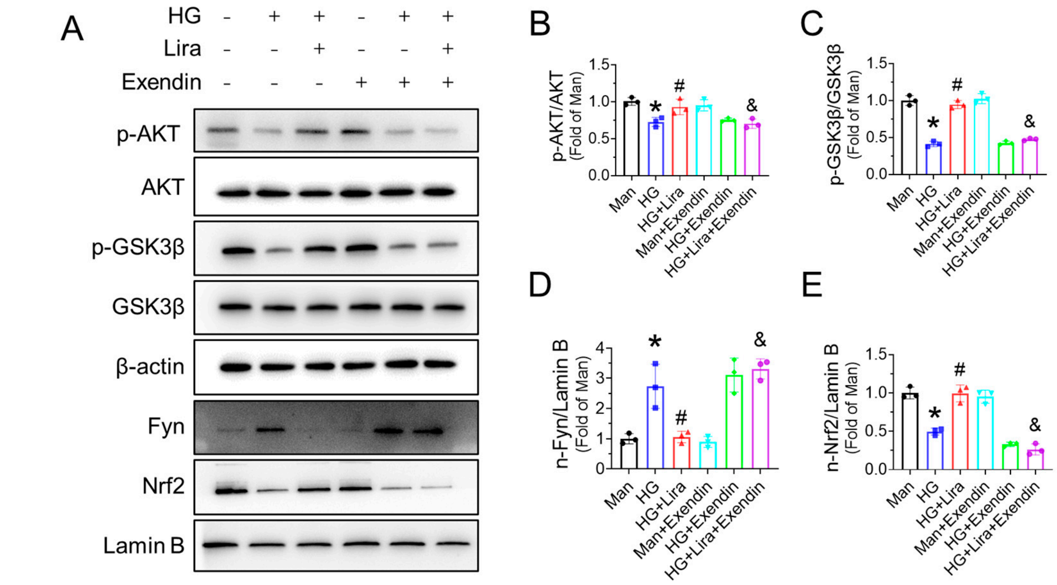

Figure S6. Exendin eliminated the effects of liraglutide on AKT pathway

activation in HG-treated EPCs.** The effects of Exendin on the activation of AKT pathway in HG-treated EPCs in the presence or absence of liraglutide were determined by western blot (A). The phosphorylation of AKT (B), the phosphorylation of GSK3β (C), decrease of nuclear Fyn content (D), and nuclear accumulation of Nrf2 (E) were quantified using Imagequant. β-Actin was used as loading control cytoplasmic proteins detecting, and Lamin B was used as loading control for nuclear proteins detecting. n = 3 per group. *P < 0.05 vs. mannitol group, #P < 0.05 vs. HG group, & P < 0.05 vs. HG+Lira group.

Figure S7

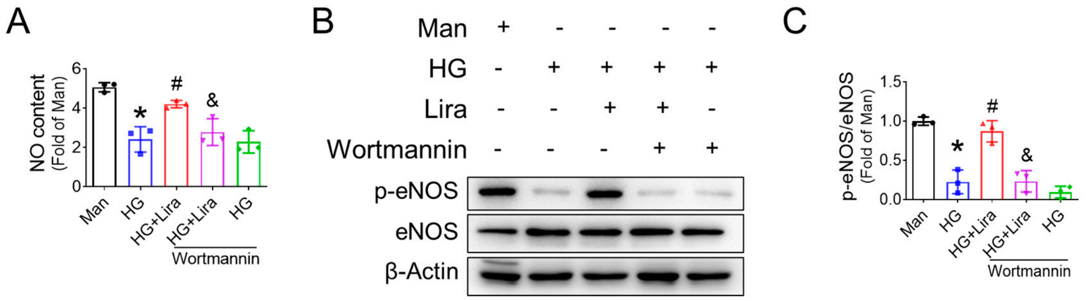

Figure S7. Wortmannin abrogated the effect of liraglutide in restoring NO bioavailability in HG-treated EPCs. NO content in the supernatant (A) of EPCs from different groups were detected using NO Assay kit. The phosphorylation of eNOS at Ser1177 was detected by Western blot (B) and quantified using Imagequant (C). Data shown in graphs represent the mean ± S.D. n = 3 per group. β-Actin was used as loading control. *P < 0.05 vs. Mannitol group; #P < 0.05 vs. HG group, & P < 0.05 vs. HG+Lira group.
